# Investigating the relationship between climate change and malaria in West Africa using the Hydrology, Entomology and Malaria Transmission Simulator (HYDREMATS)

**DOI:** 10.1186/1475-2875-13-S1-P94

**Published:** 2014-09-22

**Authors:** Teresa Yamana, Elfatih Eltahir

**Affiliations:** 1 Massachusetts Institute of Technology, Cambridge, MA, USA

## Background

The changing temperature and rainfall patterns associated with climate change are expected to alter the distribution of environmental suitability for malaria transmission in West Africa.

## Materials and methods

We use a mechanistic model of disease transmission to investigate the effects of climate change on village scale hydrology, entomology, and disease transmission. This highly detailed model explicitly simulates water pools that serve as mosquito breeding sites, the life cycle of individual mosquito agents, and the transmission of the malaria parasite between human agents.

## Results

We simulate current malaria conditions ranging from the very low transmission region bordering the Sahara to the higher transmission Savanna zones, focusing on areas with high sensitivity to increases in vectorial capacity. We then consider predictions of future climate, and assess the impact these changes would on malaria transmission.

## Conclusions

The use of this mechanistic model allows us to translate projected changes in temperature and rainfall into changes in vectorial capacity and malaria transmission rates.

